# The Pattern of Road Traffic Crashes in South East Iran

**DOI:** 10.5539/gjhs.v8n9p149

**Published:** 2015-10-29

**Authors:** Mahdieh Rad, Alexandra LC. Martiniuk, Alireza Ansari-Moghaddam, Mahdi Mohammadi, Fariborz Rashedi, Ardavan Ghasemi

**Affiliations:** 1 Health Promotion Research Center, Zahedan University of Medical Sciences, Zahedan, Iran; 2 The George Institute for Global Health at the University of Sydney, Australia, Dalla Lana; 3 School of Public Health, University of Toronto; Sunnybrook Health Sciences Centre, Toronto, Canada

**Keywords:** causal factors, human factors, injury, road traffic crash, South-East of Iran

## Abstract

**Background:**

In the present study, the epidemiologic aspects of road traffic crashes in South East of Iran are described.

**Methods:**

This cross-sectional study included the profile of 2398 motor vehicle crashes recorded in the police office in one Year in South East of Iran. Data collected included: demographics, the type of crash, type of involved vehicle, location of crash and factors contributing to the crash. Descriptive statistics were used for data analysis.

**Results:**

Collisions with other vehicles or objects contributed the highest proportion (62.4%) of motor vehicle crashes. Human factors including careless driving, violating traffic laws, speeding, and sleep deprivation/fatigue were the most important causal factors accounting for 90% of road crashes. Data shows that 41% of drivers were not using a seat belt at the time of crash. One- third of the crashes resulted in injury (25%) or death (5%).

**Conclusions:**

Reckless driving such as speeding and violation of traffic laws are major risk factors for crashes in the South East of Iran. This highlights the need for education along with traffic law enforcement to reduce motor vehicle crashes in future.

## 1. Introduction

Motor vehicle crashes (MVC) have emerged as a major public health burden, causing 20 to 50 million injuries and disabilities and nearly 1.2 million deaths per annum globally (Rezaei, Arab, Karami Matin, & Akbari Sari, 2014). Currently, road traffic injuries are the ninth leading contributor to the global burden of disease across all age groups (World Health Organization [WHO], 2015; Peden, 2005; Geziary, El-Sayed, Hussain, & Sakr, 2004; Mohan, 2008; Peden et al., 2004). However, it has been anticipated that if the present trend of MVCs continues without adequate intervention, it will rank as the 7th leading cause of death by 2030 (WHO, 2015). According to recent estimates, the annual number of deaths and disability from road traffic crashes worldwide will rise by 65% over the next decade, and in less developed countries the increase will be even greater (up to 80%) (Peden, 2005; Geziary et al., 2004).

Importantly, evidence suggests that the current and projected burden of road traffic deaths varies considerably by country and by regions within countries, with a disproportionate number occurring in low and middle income countries. For example, about 90% of the world’s fatalities on the road traffic crashes occur in developing nations, while those countries have nearly half of the world’s vehicles (WHO, 2015). In 2010, the highest mortality rates from motor vehicle crashes were reported in African region (24.1 per 100 000), whereas the mortality rates were lowest in high income countries (8.7 per 100 000). In comparison, in low- and middle-income countries, the mortality rates were high as18.3 and 20.1 per 100 000, respectively (WHO, 2015).

The pattern of MVCs also differs by age and sex such that the majority of crashes involve men aged 15-44 years, the most economically productive age group (Geziary et al., 2004). Moreover, MVCs are now the first leading cause of death among those aged 15-29 years and responsible for approximately 3 percent of Gross National Product (GNP) of the Governments (WHO, 2015).

Multiple epidemiological studies have examined modifiable factors contributing to MVCs including human, environmental, and vehicle-related variables. The most frequent causes of MVCs in developing countries that elucidated from previous studies include: reckless driving (i.e. speeding, violation of traffic signals, improper overtaking, driving too close to vehicle ahead, and intrusion of driving line), unsafe vehicles, and unsafe road design (Tavakoli Kashani, Shariat-Mohaymany, & Ranjbari, 2012; Salamati, Moradi, Soori, Amiri, & Soltani, 2015; Mitchell, Senserrick, Bambach, & Mattos, 2015; B. Lankarani et al., 2014; Shawky, Hassan, Garib, & Al-Harthei, 2015; Feng, Li, Ci, & Zhang, 2015; Rakotonirainy, Chen, Scott-Parker, Wai Loke, & Krishnaswamy, 2015; Barengo, Mkamba, Mshana, & Miettola, 2006; Mishra, M. Sinha, Sukhla, & A. Sinha, 2010; Agnihotri & Joshi, 2006).

MVCs is also a major public health concern in Iran due to several factors including rapid motorization, increase use of private vehicles rather than public transport, tendency of risk driving among youths, low gas price as well as nonstandard safety designs. (B. Lankarani et al., 2014) For example, there have been 17 million registered vehicles (roughly one vehicle for every four persons) in Iran in 2008. Additionally, it has been anticipated to increase this figure annually by 1 to 1.5 million (Hasanzadeh, Moradinazar, Najafi, & Ahmadi-Jouybary, 2014). Consequently, road traffic injuries are now the second highest cause of mortality and largest cause of years of life lost (YLL) in Iran after cardiovascular diseases accounting for almost 5 percent of GNP in the country (Rezaei et al., 2014).

Available data revealed that more than 20,000 people (i.e. 3 persons per hour) mostly the young and children lost their lives prematurely and about 280,000 were injured in 2007. Statistically, death rate caused by MVCs has declined during past decade from 38 in 2004 to 31 per 100,000 populations in 2011. Nevertheless, there has been an increase in mortality rate from 51 to 65 cases per 1000 accidents from 2004 to 2011 respectively (Bahadorimonfared et al., 2013). In addition, some population-based studies (Saadat, & Soori, 2011; Ma, Li, Zhou, Duan, & Bishai, 2012) showed that true number of MVCs might be several times higher than rates reported by surveillance system. Then, an annual average of more than 20,000 deaths is significant and should be given serious attentions (Bahadorimonfared et al., 2013).

Furthermore, a recent study demonstrated that road traffic crashes injury with an incidence rate of 308 per 100,000 is the most common risk factor for injury mirrors in South-Eastern Iran, Sistan & Bluchestan province one of the less developed part of the country (Ansari-Moghaddam et al., 2012). Nevertheless, there is inadequate epidemiological data to explain the pattern of road traffic injury in Iran, especially in deprived regions with inadequate infrastructures. Therefore, the current study aimed to identify the epidemiologic aspects of road traffic crashes in Sistan & Baluchestan Province.

## 2. Subjects and Methods

### 2.1 Sistan & Baluchestan Province

This cross-sectional study involved review of all 2398 motor vehicle crashes (MVC) recorded in the police office from 21 March 2009 to 20 March 2010 (1388 Hijri-Shamsi: One Iranian Year) in Sistan & Baluchestan province, South-East of Iran. This province is one of the largest provinces in the country with an area of about 180 000 km^2^ and a population of 2.4 million. Population concentration is very scattered with the average distance of 65 Kilometers between the major cities. Hot-dry weather in the north and most parts of the west (Kavir-e-Loot Desert), and hot-humid weather in the south (Oman Sea) makes the area one of the driest regions in Iran with the majority of days being sunny and clear. Due to limited flights from the capital city in the north, Zahedan City, to Chabahar in the south, the main transport route for passengers is roads with 636 Km driving. Cities are connected by main roads and rural areas are connected to the main roads through side roads. Most roads are undivided two-way with one narrow lane for each side (Ansari-Moghaddam et al., 2012; Piran, 2000).

### 2.2 Subjects

All persons involved in a motor vehicle crash and registered in routinely collected police data were included. Trained and experienced officers were responsible for completing registration forms used nationally for all crashes individually at the place of crash occurrence on the roads. The definition of MVC was any type of crash occurring on the road between two or more objects where at least one of the objects involved was a moving vehicle. Also, non-collision crashes such as falling, roll-overing, overturning, or fire explosion included. Reporting and definitions are standard across all police offices. Injury definition for the purpose of this study was an individual requiring any medical attention.

### 2.3 Procedure

Data collected included: demographics, the type of crash, type of involved vehicle, location of crash and factors contributing to the crash. This study was approved by the research ethics committee of Zahedan University of Medical Sciences, Iran. Furthermore, authors were granted permission from the police authorities to obtain relevant data from these files. The data required for this study was extracted from the full police datasets by two trained undergraduate student researchers with the assistance of police record-keeper officers under the supervision of the lead investigators. No names or identifying information were extracted from the police data. Descriptive statistics were completed using SPSS 15.

## 3. Results

**Figure 1:**
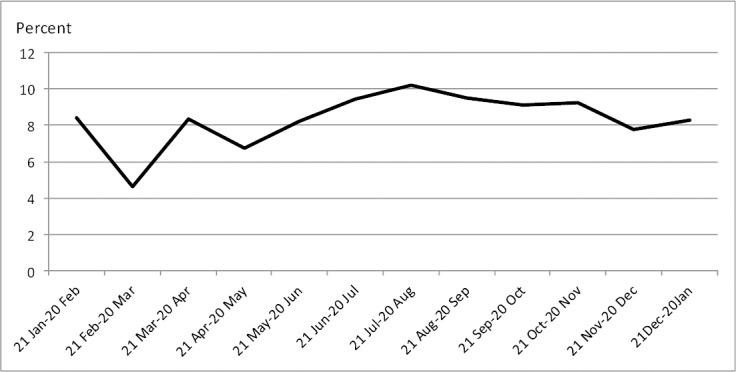
Crashes by middle of month of year

During one Iranian year, a total of 2398 road traffic crashes were recorded in Sistan & Baluchestan province. Males represented 99% of all involved drivers. The mean age of drivers was 34 (95% CI: 33-35) years and adults between the age 15 and 45 years represented the majority of (83 %) drivers. As Figure 1 shows, the distribution of crashes varied by months of year from 112 (4.6%) to 247 (10.2%). The maximum number of MVCs cases (10.2% of all MVCs) reported was in the month of Mordad (21 Jul- 20 Aug.) which is the middle of summer holiday. The least incidents (4.6% of all MCVs) occurred during the last month of the Iranian year-Esfand (19 Feb-20 Mar).

**Figure 2:**
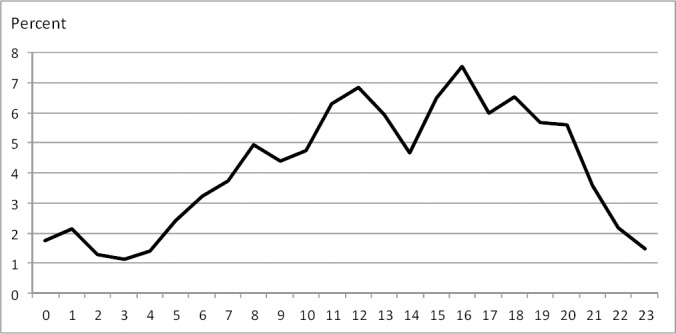
Crashes by hours of day

According to Figure 2, the majority of crashes (n = 1781; 76%) occurred between 08:00 to 20:00 with no evidence of heterogeneity across different months of the year, likely due to the larger number of drivers on the roads in these hours. Indeed, the most common peak crash times are 12 am, 4 and 6 pm. Most of the crashes occurred during sunny/clear days (n = 2238; 94%).

### 3.1 Location of Crash

As Table 1 indicates, the road surface type and condition was bitumen/asphalt (n=1847; 90%) and dry (n=2287; 96%) for the majority of the crashes. Additionally, 77.6% of crashes (n=796) occurred on straight and level roads with the remaining occurring on curved and level roads (n=313; 13.5%) or straight/curve and grade roads (n=197; 8.9%). Two-way, undivided roads were the location of 93% (n=1963) of all crashes. Thirty-eight percent of crashes (n=901) were reported on roads with poorly visible line markings.

**Table 1:** Location of crash

Location of crash			No (%)
Road Character		Straight and level	1796 (77.6)
		Curve and level	313 (13.5)
		Straight and grade	84 (3.60)
		Curve and grade	122 (5.30)
Road surface type		Bitumen/asphalt	1847 (90.4)
		Gravel or dirt	197 (9.60)
Road surface condition		Dry	2287 (95.9)
		Wet	59 (2.50)
		Others (i.e. sand, mud, oil, dirt)	39 (1.60)
Environment/Weather condition		Clear	2238 (93.9)
		Cloudy/rain/snow	102 (4.30)
		Others (i.e. blowing sand)	43 (1.80)
Road Width	Main road	≤ 6 m	57 (5.10)
		6–8 m	935 (85.9)
		8–10 m	69 (6.20)
		>10 m	31 (2.80)
	Side road	≤ 5 m	127 (12.7)
		5–6 m	549 (54.9)
		6–7 m	262 (26.2)
		≥7 m	62 (6.20)
Traffic-way description		Two-way, not divided	1963 (93.3)
		Two-way, divided	88 (4.20)
		One-way	52 (2.50)
Road defects (5 common defects)		Narrow width	978 (40.7)
		Defect of vertical signs	705 (29.2)
		Defect of horizontal signs	659 (27.3)
		Lack/defect of protection	446 (18.5)
		Lack of shoulder	270 (11.2)
Broken and solid lines marking		Proper	1436 (60.0)
		Improper	621 (26.0)
		Did not have	280 (12.0)
		Did not need	59 (2.00)
Speed limit at location of crash		≤ 20 km/h	66 (3.00)
		20-40 km/h	372 (17.1)
		40-60 km/h	654 (30.0)
		60-80 km/h	317 (14.6)
		80-90 km/h	463 (21.3)
		>90 km/h	306 (14.0)

The Table 1 also displayes the speed limit at the location of the crash was 40- 60 (km/hour) in one-third (n=654) and 80-90 (km/hour) in one-fifth (n=463) of road crashes. This was followed by 20-40 (km/hour) responsible for 17.1% (n=372), 60-80 (km/hour) with 14.6% (n=317) and more than 90 (km/hour) accounting for 14% (n=306) of crashes.

Main roads and side-roads were the locations for 47.4% (n=1126) and 42.5% (n=1009) of road crashes, respectively. The majority of main roads were as wide as 6 – 8 meters (n=935; 86%) at the location of the crash. Correspondingly, the road width was 5-6 meters at 55% (n=549) of crash locations on side roads, followed by 6-7 meters (n=262; 26%) and < 5 meters (n=127; 13%).

### 3.2 Vehicles Involved

Passenger cars (n=1543; 47.2%), pick-up truck (n=762; 23.3%) and truck (n=490; 15.4%) were the most frequently involved in crashes, comprising a total of 85.9% of the reported crashes, with motorcycle accounting for 10.4% (n=339), bus/minibus 2.5% (n=83) and others (i.e. fire rescue vehicle, police vehicle, ambulance, and bicycle) 1.2% (n = 42).

### 3.3 Type of MVCs

As can be seen in Table 2, collisions with other vehicles or objects contributed the highest proportion (n = 1497; 62.4%) of road traffic crashes with remaining crash types being non-collisions such as falling, roll-overing, overturning, or fire explosion. Further analysis indicated that collisions with a single vehicle, motorcycle/bicycle and pedestrians accounted for more than 80% (1222 out of 1497) of all collision incidents. The data additionally shows that vehicle rollover was the main reason (811 out of 901; 90%) for these non-collision incidents.

**Table 2:** Type of crash

Type of crash		No (%)
Collisions with	Motorcycle or bicycle	262 (10.9)
	Single vehicle	781 (32.6)
	Multiple vehicles	96 (4.00)
	Animals	64 (2.70)
	Pedestrians	179 (7.50)
	Fixed Objects	115 (4.80)

Non collision	rollover/overturn	811 (33.8)
	Others (i.e. fire explosion)	90 (3.80)

Total		2398 (100)

### 3.4 Causes of Crashes as Reported by Police

Overall, more than one contributing factor to a crash might be reported by police. Accordingly, human factor was a causal factor in about 90% (n=2158) of all road crashes. The main human reasons for these crashes were careless driving and violating traffic laws (i.e. improper overtaking, losing control), driving fast/speeding, and sleep deprivation/fatigue, all together responsible for 94% of all human errors (Table 3). Mechanical defaults were stated to contribute to 13% (10% alone and 3% along with human errors) of all MVCs. Defective tires, brakes, and lights were reported as the main vehicle defaults related to the crash. Of those vehicles which crashed, only 6% (n=161) had ABS. Moreover, just one-third (n=986) of the vehicles were less than 5 years old. The major road factors reported by police comprised narrow width of the road (n=978; 41%), inadequate vertical (n=705; 29%) or horizontal (n=659; 27%) signs, lack of protection (n=446; 19%) and lack of road shoulder (n=270; 11%).

**Table 3:** Human Factors contributing to the crash

Human Factors contributing to the crash	No (%)
Disregarding traffic laws	1170 (49.1)
Driving fast/speeding	460 (19.3)
Disregarding traffic laws plus driving fast	446 (18.7)
Sleep deprivation & Fatigue	127 (5.30)
Disregarding traffic laws plus Sleep deprivation & Fatigue	27 (1.10)
Driver impairment	32 (1.40)
Others	118 (4.90)

### 3.5 Seat Belt Use

Data regarding seat belt use was only available for 62% of drivers who were involved in crashes. Based on the available data, 41% of drivers were not using a seat belt at the time of crash occurrence.

### 3.6 Injury and Death

One- third of the crashes resulted in injury (25%) or death (5%).

## 4. Discussion

The results of this study suggest that driver errors and negligence were major contributing factors in most crashes in the South-East of Iran. Three human factors were observed to contribute to the vast majority (more than 90%) of the road crashes in this region: violation of traffic laws, speeding and fatigue. Unsafe road conditions (i.e. narrow road width, inadequate signs) and mechanical defects of the vehicle were also contributing factors to crashes. The reproductive male age-group (15 to 45 years) bear the brunt of road traffic crashes in this study.

Globally, young men aged 15 to 44 years account for over 70% of the total years of life lost due to MVC (Mohan, 2008; Peden et al., 2004; Odero, Gamer, & Zwi, 1997). The findings of the present study confirm that traffic crashes disproportionately affect this economically active and productive age group in the South East of Iran, as has been observed in neighbouring Arabic countries (i.e. Qatar and Saudi Arabia) (Bener, 2005; Barengo et al., 2006; Yang & Kim, 2003; Forjouh, 2003; Ansari, Akhdar, Mandooran, & Moutaery, 2000). This might be explained by the greater exposure of men as drivers, compared to women as women’s mobility is traditionally restricted and men may spend substantially more times in moving vehicles than women (WHO, 2006). Therefore, efforts to prevent motor vehicle crashes in Iran, as in similar countries, should focus upon the high risk population of men aged 15 to 44 years.

The role of speed in MVCs injuries and deaths is well known (Odero, 1997; Forjouh, 2003; Afukaar, 2003; Elvik, 2012). However, the proportion of crashes attributed to high speed varies widely, from 8.5% in Kenya to 67% in Saudi Arabia (Odero et al., 1997). Speeding was also responsible for 38% of crashes (19.3% alone and 18.7% along with disregarding traffic laws) in the current study which is in line with the previous studies. Therefore, MVCs could partly reduce by observing speed limits as a study in Brazil (Forjouh, 2003) demonstrated an over 20% reduction in traffic crashes and deaths due to reducing the speed limit.

In a review, driver negligence including reckless driving, improper overtaking and disregarding traffic laws were judged as the other main contributors to crashes by police (Odero et al., 1997). Similarly, this study from the south of Iran also demonstrates that human errors including speeding and careless driving are important factors in motor vehicle crashes.

Of particular note for policy-makers was the finding from this study that vehicle condition and protective devices in crashes included in this study were below internationally accepted standard to protect occupants in a crash. For example, only 6% of involved vehicles were equipped with ABS brakes. Moreover, about two-thirds of the vehicles were more than 5 years old. In these older vehicles, protective devices (i.e. ABS brakes) are much less likely to be either available or applicable. Our findings also highlight that poor infrastructure, such as narrow roads and inadequate signals or protection on the road also is likely to synergistically contribute to road crash causalities.

There has been greater incidence of MVCs in developing countries (between 60% and 80%) during the day than night in most previous studies (Odero et al., 1997). This was also observed in the present study, with approximately 80% accidents occurring between 8:00 to 20:00. Importantly, the most common peak crash times were 12 am, 4 and 6 pm in this time period. Due to distance between cities, people try to arrive to the capital or main cities around 12 am for some administrative affairs and get back home in the afternoon. On the other hand, there is no sufficient facility in long-distance roads which makes most drivers to prefer driving during the day. As a result, the mentioned hours are rush hours for driving in the region. However, - some previous studies have demonstrated higher numbers of crashes occurring in the night-time hours (i.e. Nigeria and Tanzania) (Forjouh, 2003). This might be due to different amounts of traffic by day/night.

It has been well documented that seat belt use reduces fatalities and serious injuries by about 50% (Forjouh, 2003). However, in this study 41% of drivers did not use a seat belt at the time of crash occurrence which is higher than those figures reported in some similar studies in the country and the region (Soori, Hussain, & Razzak, 2012; Vakili, Danaei, Askarian, Palenik, & Abdollahifard, 2012). Nevertheless, it was lower than the rate reported nationally (90%) based on the WHO’s recent report (WHO, 2013). Currently in Iran, the use of seat belts for drivers and the passengers are compulsory. However, these data suggest that further encouragement and enforcement programmes are needed.

Previous studies evaluating intervention programs to increase seat-belt use, and reduce vehicle and road hazards have demonstrated benefit (Mohammadzadeh, Paravar, Mirzadeh, Mohammadzadeh & Mahdian, 2015; Yang & Kim, 2003; Forjouh, 2003). One longitudinal and interventional controlled trial study in Iran revealed that due to the implementation of new traffic laws, the mortality and non-mortality injury rates reduced to 8.7% and 33.3%, respectively. Moreover, after 6 months of intervention, the reduction on mortality and injury rates reported to 0.4 and 11.1 per 100 000 populations, respectively (Ainy et al., 2014). Similarly, an interventional study in Korea (Yang, & Kim, 2003) showed that multiple policy interventions with the involvement of related stakeholders including policymakers, police, the media and the public effectively reduced road traffic crashes and their related injuries and fatalities.

The main limitation of our study was that this information was collected for other purposes, because of this some variables (i.e opium or alcohol use, mobile phone use, sleep quality, health status of drivers and final outcome of injury) were not complete enough to include in our analyses. Another limitation was missing data for some recorded variables such as seatbelt use. Furthermore, police officers may focus their attention on identifying the individual at fault when investigating a traffic crash. This may have led to less attention paid to potential environmental risk factors for the crash. However, despite its limitations, this study provides novel data of road traffic injuries in one of the least developed provinces in Iranians information. The information could be used in structured planning towards prevention of MVCs, in particular in the regions with similar geographical conditions including central, east and south-east of the country. Additionally, the findings of the current study regarding variables with missing data (i.e. seatbelt use) are comparable with the result of previous studies in the country or Middle-East region (WHO, 2006) which indicate the fact that missing values did not affect the result considerably.

## 5. Conclusion

Road traffic injuries pose a major threat to the well-being of society, in particular young adult males in the South East of Iran. Based on the evidence presented in this paper, behavioural factors were the main risk factors for crashes. Further research in Iran could elucidate why these human errors occur while traffic and speed checks exist all around the country. Additionally, it should be clarified whether there is any relationship between violation of traffic laws with the deficiency in the legal, policy and institutional regulations.

Accordingly, a combination of increased education along with increased traffic law enforcement may help to reduce these risky driving behaviours. In particular, the education could be targeted toward reckless driving such as speeding and violation of traffic laws. Furthermore, crashes and concomitant injuries and death could be avoided with increased control of vehicle defects and hazardous road conditions.
